# Immunophenotypic changes in the tumor and tumor microenvironment during progression to multiple myeloma

**DOI:** 10.1371/journal.pgen.1011848

**Published:** 2025-10-07

**Authors:** Isabelle Bergiers, Murat Cem Köse, Sheri Skerget, Milan Malfait, Nele Fourneau, Jenna-Claire Ellis, Greet Vanhoof, Tina Smets, Bie Verbist, Dries De Maeyer, Jeroen Van Houdt, Koen Van der Borght, Raluca Verona, Bradley Heidrich, William Kurth, Michel Delforge, Nathalie Meuleman, Jan Van Droogenbroeck, Philip Vlummens, Christoph J. Heuck, Yves Beguin, Nizar Bahlis, Tineke Casneuf, Jo Caers

**Affiliations:** 1 Johnson & Johnson, Beerse, Belgium; 2 Department of Hematology, CHU of Liège, Liège, Belgium; 3 Johnson & Johnson, Spring House, Spring House, Pennsylvania, United States of America; 4 Department of Applied Mathematics, Computer Science and Statistics, University of Ghent, Ghent, Belgium; 5 Department of Orthopedic Surgery, CHU de Liège, Liège, Belgium; 6 University Hospital Leuven, Leuven, Belgium; 7 Department of Hematology, Institut Jules Bordet, Hôpital Universitaire de Bruxelles, Université Libre de Bruxelles, Brussels, Belgium; 8 Department of Haematology, AZ Sint-Jan Brugge-Oostende AV, Brugge, Belgium; 9 Department of Clinical Hematology, Ghent University Hospital, Ghent, Belgium; 10 Department of Hematology and Oncology, University of Calgary, Calgary, Alberta, Canada; National Cancer Institute, UNITED STATES OF AMERICA

## Abstract

Investigation of the cellular and molecular mechanisms of disease progression from precursor plasma cell disorders to active disease increases our understanding of multiple myeloma (MM) pathogenesis and supports the development of novel therapeutic strategies. In this analysis, single-cell RNA sequencing, surface protein profiling, and B lymphocyte antigen receptor profiling of unsorted, whole bone marrow (BM) mononuclear cell samples was used to study molecular changes in tumor cells and the tumor microenvironment (TME). A cell atlas of the BM microenvironment was generated from 123 subjects including healthy volunteers and patients with monoclonal gammopathy of unknown significance (MGUS), smoldering MM (SMM), and MM. These analyses revealed commonalities in molecular pathways, including MYC signaling, E2F targets and interferon alpha response, that were altered during disease progression. Evidence of early dysregulation of the immune system in MGUS and SMM, which increases and impacts many cell types as the disease progresses, was found. In parallel with disease progression, population shifts in CD8 + T cells, macrophages, and classical dendritic cells were observed, and the resulting differences in CD8 + T cells and macrophages were associated with poor overall survival outcomes. Potential ligand-receptor interactions that may play a role during the transition from precursor stages to MM were identified, along with potential biomarkers of disease progression, some of which may represent novel therapeutic targets. MIF, IL15, CD320, HGF and FAM3C were detected as potential regulators of the TME by plasma cells, while SERPINA1 and BAFF (TNFSF13B) were found to have the highest potential to contribute to the downstream changes observed between precursor stage and MM cells. These findings demonstrate that myeloma tumorigenesis is associated with dysregulation of molecular pathways driven by gradually occurring immunophenotypic changes in the tumor and TME.

**Trial registration:** This project has been registered at EudraCT (European Union Drug Regulating Authorities Clinical Trials Database) with protocol number NOPRODMMY0001 and EudraCT Number 2018-004443-23 on 12 December 2018.

## Author summary

Multiple myeloma (MM) has two distinct precursor conditions: monoclonal gammopathy of unknown significance (MGUS) and smoldering multiple myeloma (SMM). Only a subset of patients with these precursor conditions eventually progress to develop symptomatic MM, and the factors driving progression remain poorly understood. In this study, we collected bone marrow samples from healthy volunteers, and patients with MGUS, SMM, and MM at the time of diagnosis. Using single cell multi-omics, we examined cells of the tumor and tumor microenvironment (the cells surrounding the tumor) to identify the changes that occur during progression to MM, including changes in cell proportions and gene expression. We identified early dysregulation of the immune system, including increased inflammation, in MGUS which increased in SMM and MM. Further, we observed shifts towards specific CD8 + activated T cell and macrophage cell populations, and showed that patients with higher proportions of these cells at MM diagnosis were associated with adverse survival outcomes. Overall, we show that progression from precursors to MM is driven by gradually occurring changes in both the tumor and the tumor microenvironment.

## Introduction

Multiple myeloma (MM), a hematologic malignancy of bone marrow (BM) plasma cells [1], is the second most common blood cancer in the USA and is primarily considered a disease of the elderly (median age at diagnosis is 69 years) [2]. Over the past 15 years, the prognosis of patients with MM has improved due to the emergence of new therapies providing deep and durable responses [3,4]. However, these therapies are not curative, and patients will eventually relapse, underscoring the urgent need for new treatment options and a better understanding of disease progression.

MM has two premalignant stages: monoclonal gammopathy of unknown significance (MGUS), an indolent condition relatively common among elderly individuals with a rate of progression to MM of 1% per year, and smoldering multiple myeloma (SMM), with a higher rate of progression to MM of 10% per year [5]. Patients with MGUS and SMM present with clonal bone marrow plasma cells and elevated levels of monoclonal protein in the blood, but they are typically asymptomatic, lacking the MM-defining symptoms of hypercalcemia, renal impairment, anemia and bone lesions (CRAB criteria) [5,6]. Several clinical trials in MGUS and MM are investigating treatment interventions at this premalignant stage to either delay or prevent progression and to provide additional insights to inform our understanding of the underlying disease biology [7].

MM is a complex disease with high inter- and intra-patient variability. A better understanding of the interaction between the tumor, microenvironment, and intrinsic events that drive disease progression is important for the discovery of new MM treatments. MM is a heterogenous disease with multiple molecular subtypes that share a similar clinical presentation [8,9]. Previous studies have shown that genomic alterations of plasma cells characteristic of MM are detectable at MGUS and SMM stages, highlighting the contribution of tumor extrinsic factors in progression to MM [8,10–13]. Notably, changes in the BM microenvironment at different stages of MM have also been described [6,14–16]. Exploring the BM resident plasma and immune microenvironment cells, Zavidij et al. found evidence of increased natural killer cell proportions, altered chemokine receptor expression upon disease progression [15] and myeloma-associated events, such as loss of granzyme K+ (GZMK) memory cytotoxic T cells and major histocompatibility complex class II dysregulation in CD14 + monocytes, in the premalignant stages. A single-cell transcriptomic analysis of plasma cells demonstrated high variability between and within patients with MM and linked this variability with expression of known and newly identified genomic drivers of MM [14]. Spatial co-localisation of the transcription of genes involved in tumor survival and immune modulation in tumor and immune cells predicted a role for mesenchymal stromal cells in MM [17]. Although many studies highlight the value of single-cell profiling in understanding inter- and intra-patient tumor heterogeneity and the contribution of the tumor microenvironment across different stages of the disease, most have focused only on plasma cells [14,17] or the surrounding immune cells [15], have lacked inclusion of premalignant stages [17], or have been conducted on a limited number of patients [14–16,18,19].

In this study, BM samples from healthy volunteers, as well as patients with MGUS, SMM and newly diagnosed MM were profiled using multi-modal single-cell -omics techniques, including single-cell RNA sequencing (scRNA-seq), cellular indexing of transcriptomes and epitopes by antibody derived tag sequencing (ADT-seq) and B-cell receptor sequencing (BCR-seq), to investigate the status of the BM microenvironment, dysregulation of the immune system, and interaction between BM immune cell populations and tumoral plasma cells to better understand progression to MM.

## Results

### Decreases in antigen-presenting cell populations and increases in T-cell subsets were observed during MM progression

**Fig 1:**
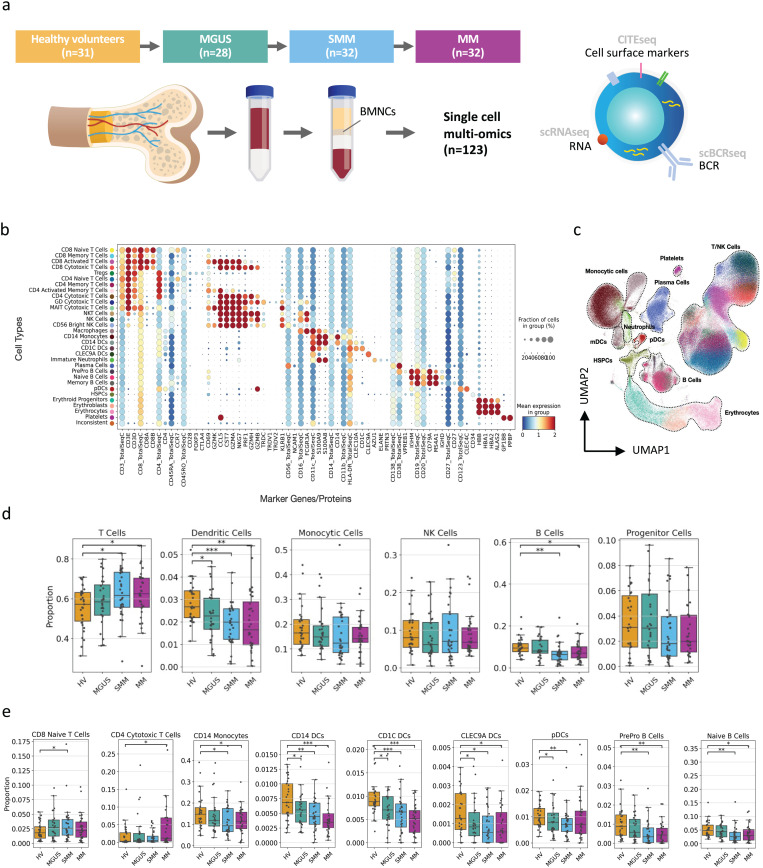
Single-cell atlas of BMNCs shows proportional changes in key immune cell populations. (A) Study design. BMNCs were collected from 123 subjects across 4 cohorts (HVs, MGUS, SMM, and newly diagnosed MM) and were further analyzed using scRNAseq, ADT-seq and scBCRseq. (B) Expression profile of marker genes/proteins for each cell type. The ‘TotalSeqC’ extension designates the proteins. (C) UMAP representation of the scRNA-seq dataset, colored by identified cell types. (D) Proportions of major cell types in each patient cohort (excluding plasma cells). (E) Proportions of minor cell types in each patient cohort (excluding plasma cells). Only cell populations where significant proportional changes were observed are displayed. For both sections (D) and (E), each dot represents an individual subject. The proportional differences between each disease state versus HVs are compared, and the significance was calculated using the Mann–Whitney U test: *p < 0.05, **p ≤ 0.005, ***p ≤ 0.0005. ADT-seq, antibody derived tag sequencing; BMNC, blood marrow mononuclear cell; DC, dendritic cell; HSPC, hematopoietic stem and progenitor cell; HV, healthy volunteers; mDC, myeloid DC; MGUS, monoclonal gammopathy of undetermined significance; MM, multiple myeloma; NK, natural killer; NKT, natural killer T cell; pDC, plasmacytoid DC; scBCRseq, single-cell B-cell receptor sequencing; scRNAseq, single-cell RNA sequencing; SMM, smoldering multiple myeloma; Treg, regulatory T cell.

The single-cell atlas of 355,857 single cells described in this study was generated using BM aspirates from 123 subjects from 4 cohorts: healthy volunteers, and patients with MGUS, SMM, and newly diagnosed MM (Fig 1A and Table 1). Multi-modal single-cell omics technologies (scRNA-, ADT- and BCR-seq) allowed the identification of the major BM cell subsets (10 major and 30 minor cell types) based on their gene and protein expression patterns (Fig 1B and 1C). The data showed a gradual decrease in B-cell and dendritic-cell (DC) populations and a gradual increase in total T cells with MGUS, SMM and MM compared with healthy volunteers (Fig 1D). At the subtype level, proportional decreases in pre-pro B cells, naive B cells, all DC subtypes and CD14 + monocytes, and proportional increases in CD8 + naïve T cells and CD4 + cytotoxic T cells (Fig 1E) were observed. Interestingly, these changes were highly variable among individuals of each cohort. Observations regarding the gradual increase in T cells and the gradual decrease in CD14 + monocytes, DCs and pre-pro B cells in this study highly correlate with the findings of Zavidij et al [15]. Additionally, a decrease in the naive B-cell population in patients with SMM (*p* < 0.005) and MM (*p* < 0.05) compared with healthy volunteers was observed.

**Table 1:** Study population characteristics.

Characteristics	HVs	MGUS	SMM	MM
Subjects, *n*	31	28	32	32
Age (years)				
Median	70	61.5	63	70
Range	59–89	38–85	50–86	45–86
Gender				
Male	16	17	20	20
Female	15	11	12	12
ISS Stage				
NA				4
I				9
II				10
III				9

HV, healthy volunteer; ISS, International Staging System; MGUS, monoclonal gammopathy of undetermined significance; MM, multiple myeloma; NA, not available; SMM, smoldering multiple myeloma.

Through Cytometry by Time Of Flight (CyTOF) analysis of the BM we further confirmed trends for decreasing proportions of B-cells, monocytes, and CD8 + naive T-cells in MGUS, SMM, and MM compared to HV in this cohort (S1 Fig). Although we did not observe a difference in overall NK cell proportion through ADTseq or CyTOF, CyTOF analysis revealed an increase in the CD56dimCD16 + CD69 + NK cell subpopulation (S2 Fig). This subpopulation was present in higher proportions in MM compared to HV (p < 0.001), MGUS (p < 0.005), and SMM (p < 0.5) and did not express exhaustion markers (PD-1-, TIM-3-, LAG-3-, TIGIT-) indicating the presence of a functional, activated NK cell subset in the BM.

### Inter-patient diversity and commonalities in transcriptional alterations observed in plasma cells across disease stages

**Fig 2:**
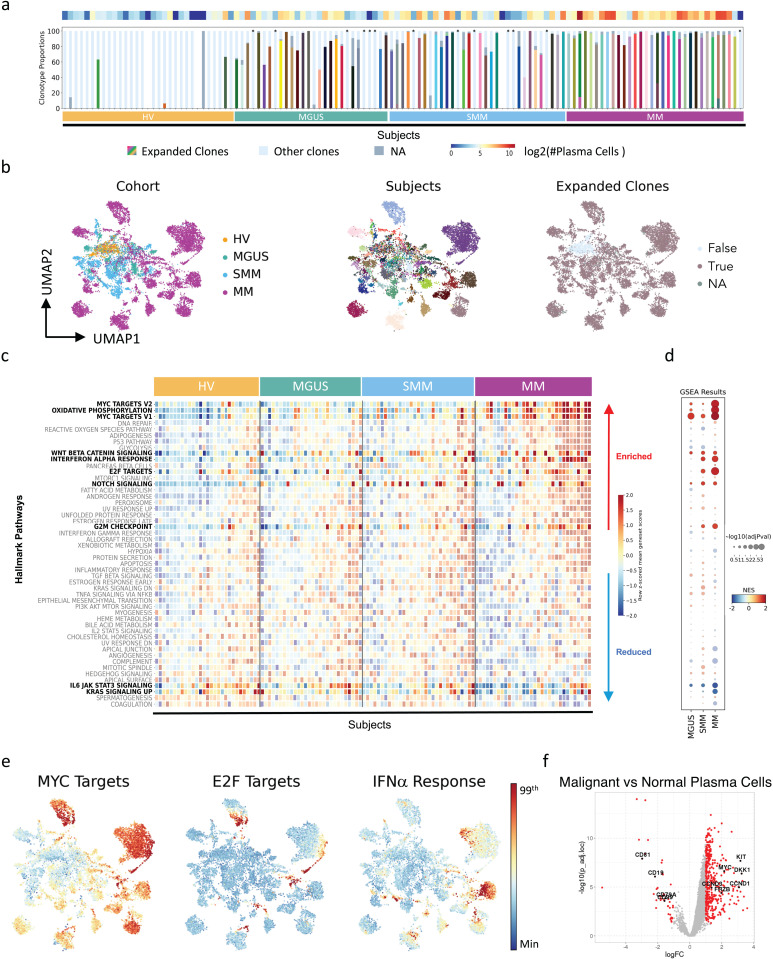
Transcriptional similarities and differences were observed in plasma cells across disease stages. (A) Cell number proportions of plasma cell clonality within subjects, obtained by BCR-seq. The heatmap on the top of the stacked bar plot shows the number of cells per sample. Expanded clones: per-subject clones with ≥ 10 cells; Other clones: identified clones with no expansion (marked with an asterisk); NA: cells with no clonal information captured by BCR-seq. (B) UMAP representation of plasma cell transcriptome, colored by cohort (left), subject (center) and expanded clonality (right) information. True: per-subject clones with at least 10 cells; False: identified clones with no expansion; NA: cells with no clonal information captured by BCR-seq. (C) Within and between cohort diversity is represented by hallmark gene set scores, where the x-axis is categorized by cohort and sorted based on mean gene set scores, whereas the y-axis is sorted by mean gene set scores in MM subjects. (D) GSEA results where each disease state is contrasted with HVs. Bolded gene sets highlighted in both panels (C) and (D) were significant in MGUS, SMM, and/or MM, and show gradual divergence at both patient and cohort level. (E) UMAP representation of transcriptome of plasma cells colored by gene set scores of hallmark gene sets enriched in precursor stages. The figure colors range from minimum value (blue) to the 99^th^ percentile of the value distribution (red). (F) Differential expression results of the contrast diseased (MGUS, SMM, MM) versus non-diseased (HVs) plasma cells. Points colored in red represent significantly down- (negative logFC) and upregulated (positive logFC) genes. Significantly down- and upregulated genes were identified based on adjusted *p* value < 0.05, absolute value of logFC > 1, and logCPM > 1. BCR-seq, B-cell receptor sequencing; GSEA, gene set enrichment analysis; HV, healthy volunteers; IFN, interferon; logCPM; log counts per million; logFC, log-fold change; MGUS, monoclonal gammopathy of undetermined significance; MM, multiple myeloma; NA, not available; NES, normalized enrichment score; SMM, smoldering multiple myeloma.NES, normalized enrichment score; SMM, smoldering multiple myeloma.

The dataset included the transcriptomes of 20,759 single plasma cells from 121 subjects (2 subjects were excluded [1 healthy volunteer and 1 patient with SMM] as no plasma cells were isolated). As expected, expansion of plasma cells was generally not found in healthy volunteers (except in 2 subjects). In 1 subject, the number of clonal cells was low (*n* = 15), whereas in the second subject, a considerably higher number of clonal cells (*n* = 130) was observed, potentially due to acute infection or other immune reaction. Samples where no clonal expansion was detected (Fig 2A, denoted by asterisks) may have had a low number of plasma cells, or sampling was performed outside of the tumor area. In patient samples, 13 of 92 (14.1%) showed no clonal expansion (Fig 2A, denoted by asterisks). A low number or lack of plasma cells may be due to the rarity of plasma cells or sampling at a location outside the tumor area.

Unsupervised dimensionality reduction revealed patterns of both transcriptional heterogeneity and similarity (Fig 2B). Non-clonal plasma cells (light blue, right panel) localized in the center, independently from patient subgroups (Fig 2B, left and right panels). In contrast, clonal cells (Fig 2B, right panel, light brown) of patients with MGUS (Fig 2B, left panel, green) localized close to those of healthy volunteers (orange), while clusters of cells from MM patients (magenta) predominantly localized on the periphery. Notably, cells of individual patients clustered together and away from those of other patients (Fig 2B, center panel), illustrating the gradual divergence of MGUS, SMM and MM plasma cell transcriptomes. Further, subclusters of plasma cells were observed for a few patients, which indicates inter- and intra-patient diversity of transcriptomes.

Plasma cell mean gene set scores calculated at the subject level (Fig 2C) and pathway enrichment analysis per cohort (Fig 2D and S1 File) revealed the presence of both intra-cohort heterogeneity and similarity. MYC signaling (MGUS, *p* < 0.05) [20], cell cycle-related E2F targets (SMM, *p* < 0.05) and interferon alpha (IFNα) response (SMM, *p* < 0.05) pathways were enriched early and increased with MM progression (Fig 2E). Oxidative phosphorylation (*p* < 0.005) [21] and G2M checkpoint (*p* < 0.05) pathways were significantly enriched only in patients with MM, while a decrease was observed in IL6/JAK/STAT3 (*p* < 0.05) signaling (led mostly by negative pathway regulators such as CD38 or PTPN1 [S1 File]), in KRAS signaling (*p* < 0.05) (including KRAS activators, while conversely there was high KRAS expression in MM patients) and in complement (*p* < 0.05) pathways. Downregulation of the KRAS signaling pathway with disease progression may represent a negative feedback loop, where genes within the KRAS pathway are downregulated due to high expression or constitutive activation of KRAS. Using per-sample gene set scoring, despite heterogeneity among subjects, the alterations in these pathways could also be observed at the patient level. Notably, although WNT signaling [22] and NOTCH signaling [23] did not meet the significance threshold, these pathways showed positive enrichment scores in some subjects with MGUS, SMM and MM.

Malignant plasma cells from patients with MGUS, SMM, or MM (expanded clones) compared with normal plasma cells of all subjects (non-expanded clones) showed 439 significantly differentially expressed genes (*p* < 0.05, |logFC| > 1, logCPM > 1), including upregulation of the known MM markers [6,8,24] FRZB, DKK1, MYC, CCND1 and CCND3 and the proto-oncogene KIT [25], and downregulation of the B-cell antigens CD27, CD19 and CD79A and the tumor suppressor genes CD81 [26] and CD99 [27] (Fig 2F and S2 File).

### Dysregulation of inflammatory response is initiated early and is gradually detected across the tumor microenvironment

**Fig 3:**
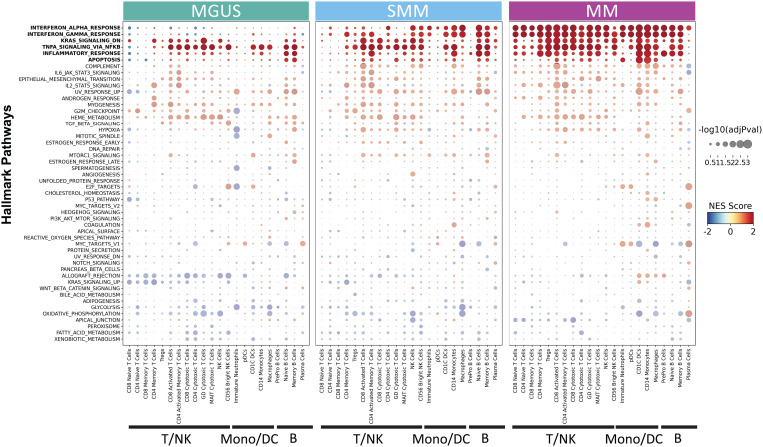
Proportional and transcriptional changes in the immune microenvironment showed early dysregulation of inflammatory response. GSEA results of hallmark gene sets where the expression profile of each disease state is contrasted by HVs per immune cell type. On the x-axis, the cell types are grouped by their corresponding major cell-type category. The y-axis is sorted based on average NES score in MM. Gene sets, enriched along disease progression, are highlighted at the top. DC, dendritic cell; GSEA, gene set enrichment analysis; HV, healthy volunteers; MGUS, monoclonal gammopathy of undetermined significance; MM, multiple myeloma; NES, normalized enrichment score; SMM, smoldering MM.

Using muscat and fgsea, activation of numerous inflammatory pathways, such as interferon gamma (IFNγ), IFNα, tumor necrosis factor alpha (TNFα), KRAS, inflammatory response and apoptosis were observed in patients at each disease stage versus healthy volunteers (Fig 3 and S3–S5 Files). Dysregulation of the inflammatory response was observed in the microenvironment of patients with MGUS, with high TNFα signaling activity in the majority of cell types. With disease progression, further dysregulation of inflammatory pathways was detected across BM cell types. Activation of IFNα, IFNγ, KRAS and apoptosis pathways was rarely observed in the MGUS stage while it was observed in multiple immune cell types in the SMM and MM stages. Interestingly, IFN pathway changes were highly enriched in all cell types in the tumor microenvironment in the MM stage, particularly IFNα in plasma cells.

### CD8 T-cell pre-dysfunctionality observed with disease progression

**Fig 4:**
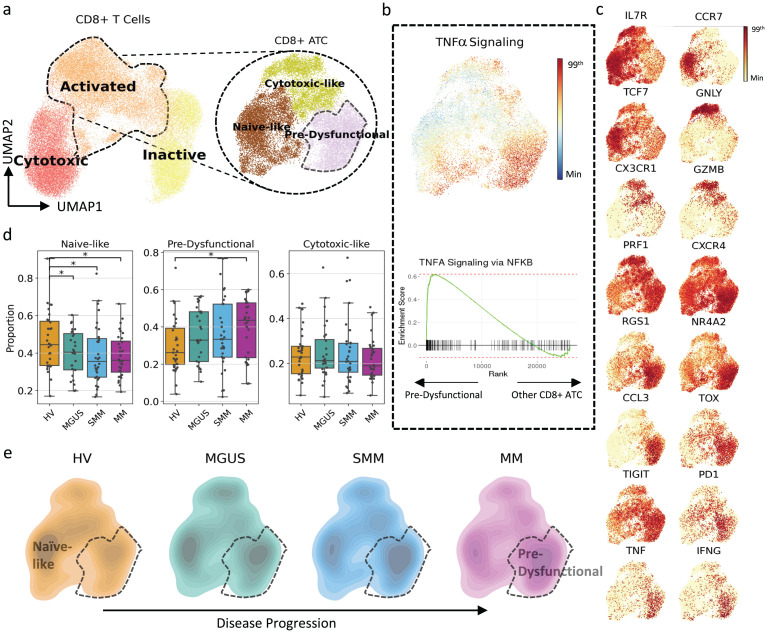
Increase in pre-dysfunctional phenotype within CD8 + ATCs observed during disease progression. (A) UMAP representation of the transcriptome of CD8 + T cells and a separate UMAP including only CD8 + ATCs each colored by cell groups in per-cell-type analyses. (B) Gene set scores and GSEA plots of TNFα signaling highlighting enrichment of TNFα signaling in pre-dysfunctional CD8 + ATCs. The figure colors range from minimum value (blue) to the 99^th^ percentile of the value distribution (red). In the GSEA analysis, the gene expression levels of pre-dysfunctional cells are compared against other CD8 + ATCs. (C) Gene expression profiles of marker genes define CD8 + ATC subtypes. The figure colors range from minimum value (yellow) to the 99^th^ percentile of the value distribution (red). (D) Proportional differences among cohorts for CD8 + ATC subtypes. Each dot represents an individual subject. The proportional differences between each disease state versus HVs are compared, and the significance was calculated using the Mann–Whitney U test: *p < 0.05, **p ≤ 0.005, ***p ≤ 0.0005. (E) Density plots showing the transcriptomic landscape of CD8 + ATCs per cohort. The highlighted area represents the region where the pre-dysfunctional cells reside. A transcriptional shift with disease progression was observed. ATCs, activated T cells; GSEA, gene set enrichment analysis; HV, healthy volunteers; MGUS, monoclonal gammopathy of undetermined significance; MM, multiple myeloma; SMM, smoldering MM; TNF, tumor necrosis factor.

**Fig 5:**
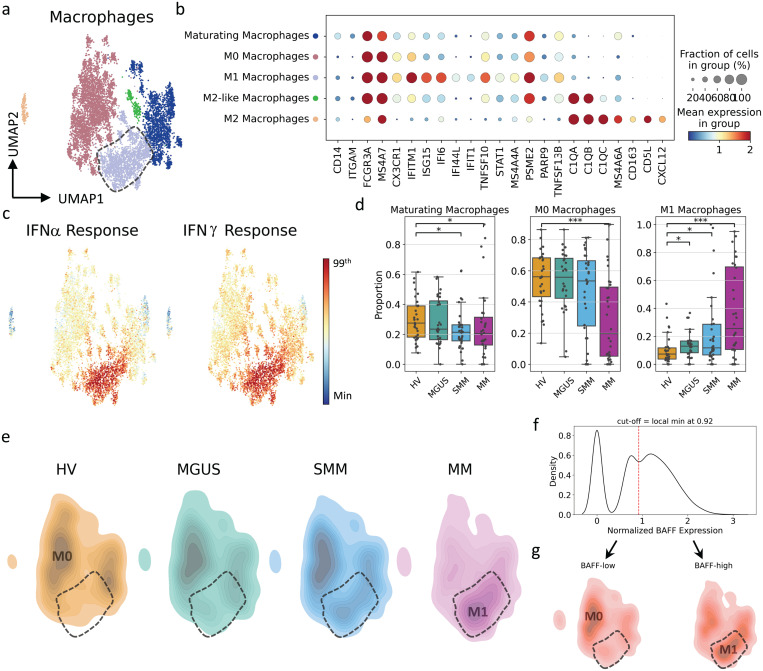
Macrophages polarized towards an M1 phenotype during myeloma disease progression. (A) UMAP representation of transcriptome of the macrophage population, colored by subtype annotations. (B) Expression profiles of marker genes that define macrophage subpopulations. (C) Gene set scores of IFNα and IFNγ highlight IFN-responsive regions in macrophages. The figure colors range from minimum value (blue) to the 99^th^ percentile of the value distribution (red). (D) Proportional differences among cohorts for macrophage subtypes. Each dot represents an individual subject. The proportional differences between each disease state versus HVs are compared, and the significance was calculated using the Mann–Whitney U test: *p < 0.05, **p ≤ 0.005, ***p ≤ 0.0005. (E) Density plots showing the transcriptomic landscape of macrophages per cohort. The highlighted area represents the region where M1 macrophages reside. (F) The distribution of BAFF gene expression within the macrophage population. The cut-off value was defined by the local minima at 0.92 to separate BAFF-low and BAFF-high populations. (G) Density plots of the BAFF-low and -high populations, showing dense regions on transcriptomic landscape for both categories. The highlighted area represents the region where the M1 macrophages reside. HV, healthy volunteers; IFN, interferon; MGUS, monoclonal gammopathy of undetermined significance; MM, multiple myeloma; SMM, smoldering MM.

**Fig 6:**
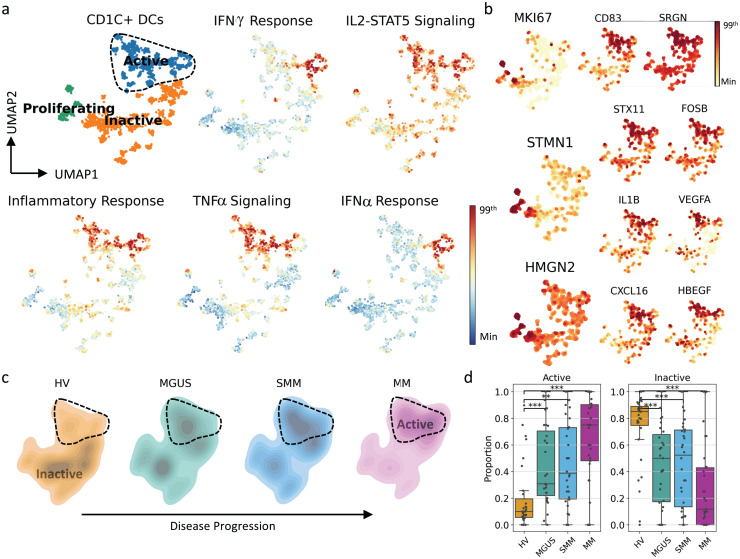
CD1C + DCs activation and maturity increased in parallel with disease progression. (A) UMAP representation of transcriptome of the CD1C + DC population colored by CD1C + DC subtypes and gene set scores of various inflammation-related pathways. The figure colors range from minimum value (blue) to the 99^th^ percentile of the value distribution (red). (B) Expression profiles of marker genes define CD1C + DC subpopulations. The figure colors range from minimum value (yellow) to the 99^th^ percentile of the value distribution (red). (C) Density plots showing transcriptomic landscape of CD1C + DCs per cohort. Highlighted area represents the region where the activated CD1C + DCs reside. (D) Proportional differences among cohorts for CD1C + DC subtypes. Each dot represents an individual subject. The proportional differences between each disease state versus HVs are compared, and the significance was calculated using the Mann–Whitney U test: *p < 0.05, **p ≤ 0.005, ***p ≤ 0.0005. DC, dendritic cell; HV, healthy volunteers; IFN, interferon; MGUS, monoclonal gammopathy of undetermined significance; MM, multiple myeloma; SMM, smoldering MM; TNF, tumor necrosis factor.

By further comparing MGUS, SMM and MM cell proportions across the premalignant to MM spectrum with that of healthy volunteers, significant population shifts in CD8 + activated T cells (ATC), macrophages and CD1C + DCs were found (Figs 4–6).

CD8 + ATC can be identified within CD8 + T cells as an intermediate state in the trajectory towards a GZMB+PRF1 + cytotoxic state by their expression of activation markers GZMK, CD69 and CCL4 (Fig 4A). CD8 + ATC were further subtyped by Leiden clustering into 3 subpopulations forming a trajectory from a naive-like population (characterized by the expression of IL7R, CCR7 and TCF7) over a cytotoxic-like population (specified by GNLY, CX3CR1, GZMB and PRF1) towards a highly active ‘pre-dysfunctional’ population (Fig 4A and 4C).

A gradual shift tracks with the disease progression stages from the naive-like phenotype towards the pre-dysfunctional phenotype with a significant (*p* < 0.05) increase in the latter observed by proportional analysis in MM compared to healthy volunteers (Fig 4D and 4E). This phenotype is identified based on the high expression of CXCR4, CCL3 and exhaustion-associated genes RGS1 [28] and NR4A2 [29], expression of some but not all the exhaustion markers [30,31], TOX, TIGIT and PD1 at low levels, expression of the pro-inflammatory cytokine TNF, and their highly active TNF response (Fig 4B and 4C). Moreover, gene set enrichment analysis demonstrated downregulation of translational and metabolic mechanisms, including translation elongation, translation initiation, oxidative phosphorylation and protein localization to endoplasmic reticulum and cellular respiration in this population (S3 Fig). These changes have been shown to be associated with increased exhaustion in CD8 + T cells [32,33], while recently, Yan et al. [33] demonstrated the rapid decrease in translational and metabolic pathways in the pre-dysfunctional state along T-cell differentiation towards exhaustion.

### Macrophages polarize towards an M1 phenotype with increased BAFF expression with disease progression

Similarly, the macrophage population subclustered by Leiden clustering showed 5 distinct subpopulations annotated based on their gene expression profile (Fig 5A and 5B). The “M0 macrophage” state is characterized by the expression of macrophage markers FCGR3A and MS4A7 while it lacks the expression of CD14 as well as M1 and M2 markers (Fig 5B). “Maturing macrophages” show low expression of CD14 and MS4A7 (Fig 5B). “M1 macrophages” exhibit high IFN response expression (Figs 5C and S4) and expressed IFN-related genes, including IFI-6, IFI44L, IFIT1, TNFSF10 and STAT1 (Fig 5B). “M2 macrophages” are characterized by the expression of the C1Q gene family and M2 markers CD163, CD5L and CXCL12 (Fig 5B). And lastly, the “M2-like macrophages” lack most of the M2 markers, and exclusively expressed C1Q family genes (Figs 5B, 5C, and S4, and S2 File).

During disease progression, a differentiation of M0 and maturating macrophages towards an M1 phenotype was observed (Fig 5E). A proportional analysis demonstrated an inverse relation between M0 and maturating macrophages versus the M1 phenotype. Compared with healthy volunteers, there was a significant (*p* < 0.0005) decrease in M0 macrophages and an increase in M1 macrophages in patients with MM. Moreover, a significant (*p* < 0.05) decrease in maturating macrophages was observed in patients with SMM and MM, while a significant (*p* < 0.05) increase in M1 macrophages was observed in patients with MGUS and SMM (Fig 5D). Notably, this shift was not observed for the M2 phenotype, as the proportion of M2 macrophages did not differ significantly among disease categories (S3A Fig).

These findings suggest that at early stages of MM and in the active disease stage, the balance between M1 and M2 macrophages shifts towards the M1 phenotype. In line with this population shift, elevated expression of BAFF (TNFSF13B) was also observed. BAFF has been shown to interact with B-cell maturation antigen, which is known to enhance the growth and survival of plasma cells [34,35]. To illustrate elevated BAFF expression, macrophages were divided into BAFF-low and BAFF-high groups based on the local minimum of 0.92 from the distribution of BAFF expression levels (Fig 5F). The data showed that the BAFF-high region highly overlapped with M1 macrophages, and the BAFF-low region highly overlapped with M0 macrophages (Fig 5G).

### CD1C + DCs exhibit an active and pro-inflammatory phenotype with disease progression

CD1C + DCs were identified by the protein expression of HLA-DR as well as CLEC10A and CD1C gene expression. After clustering, 3 subtypes of CD1C + DCs were identified and designated as active, proliferating and inactive (Fig 6A).

The “active” group includes cells expressing CD83, SRGN, STX11 (Fig 6B) and NR4A family genes, activator protein 1-related genes FOSB and JUNB and anti-apoptotic genes MCL1 and CDKN1A and show a more mature and active phenotype (S4B Fig). In this population, activation of inflammatory pathways, including inflammatory response, TNFα signaling, IFNα response, IFNγ response, and IL2-STAT5 signaling, as well as T-cell activation pathways were observed (Figs 6A and S6). In addition to this highly inflammatory phenotype, expression of other pro-inflammatory genes, such as IL1B [36], CXCL16 [37,38], VEGFA [39] and HBEGF [40] (Fig 6B), which can facilitate tumor progression and survival, were also observed. The “proliferating” subtype, on the other hand, consists of a small number of cells (*n* = 167) with high expression of cell cycle-related genes MKI67*,* STMN1 and HMGN2, as well as similar gene expression levels for the marker genes used to define the active subtype (S5B Fig). Finally, the “inactive” CD1C + DCs show no/low proliferation or activation marker gene expression (Fig 6B).

When assessing phenotypic changes in CD1C + DCs, a population shift from inactive to active CD1C + DCs was observed with disease progression (Fig 6C and 6D). Similar observations were made for CD14 + DCs (S5C and S7 Figs).

Taken together, these findings suggest that there is increased maturation and activation of classical DCs (cDCs, CD1C+ and CD14 + DCs) along disease progression.

### External public datasets validate shifts in immune populations

To validate these observed shifts in immune populations and investigate their relationship with PFS and OS, external datasets from similar studies were used. The de Jong et al. dataset allowed us to evaluate the transcriptional differences in cDC and CD8 + ATC populations between healthy volunteers and MM. For both cDC and CD8 + ATC populations, similar trends were observed (S8 Fig). Within the cDC population, 2 clusters were identified among which 1 (cluster 1, S8A Fig) showed increased TNFα signaling, IFNα response, and IFNγ response as well as higher levels of DC activation markers (S8A Fig) similar to the ‘active’ populations identified above (Figs 6 and S5C). Although not significant (possibly due to the low number of samples), higher proportions of that cluster were observed in MM. Similarly, within the CD8 + ATC population, 2 clusters were identified among which 1 (cluster 0, S8B Fig) showed increased TNFα signaling and higher levels of activated pre-dysfunctional markers (S8B Fig). Higher proportions of this subgroup were observed in patients with MM compared with healthy volunteers similar to results presented in Fig 4.

For macrophages, similar findings were observed (S9 Fig). Two clusters were identified: cluster 1 showed increased IFN signaling and higher levels of M1 markers. This cluster was identified as M1 macrophages. Cluster 0 showed expression of the macrophage markers FCGR3A (CD16) and MS4A7 and lacked the M1 and M2 markers as well as CD14. Therefore, this cluster was annotated as M0 macrophages. The proportional analysis showed a significant population shift from M0 to M1 macrophages from healthy volunteers to patients with MM. Moreover, when BAFF expression was categorized into BAFF-low and -high categories based on the median cut-off at 0.68, there was an association between a high BAFF expression and M1 polarization.

### Population shifts in CD8 + T cells and macrophages are associated with poor overall survival in the CoMMpass dataset

The CoMMpass immune dataset does not include healthy volunteers, MGUS or SMM samples but is instead composed of samples collected from newly diagnosed MM patients with accompanying metadata. Thus, this dataset was used to evaluate whether differences in the identified population shifts were related to time to event outcomes (PFS and OS). For CD8 + ATCs, macrophages and cDCs, similar transcriptomic findings were observed between the current study and the CoMMpass dataset. Within cDCs, 2 clusters were identified: cluster 1 showed increased TNFα signaling and higher levels of activated DCs (S10A Fig). Similarly, within CD8 + ATCs, 3 clusters were identified, with the cluster showing increased TNFα signaling and higher levels of activated pre-dysfunctional CD8 + T-cell markers considered pre-dysfunctional (S10B Fig). For macrophages, all the subtypes identified in this study were also detected in the CoMMpass dataset, and increased BAFF expression in the M1 phenotype could be observed (S11 Fig).

**Fig 7:**
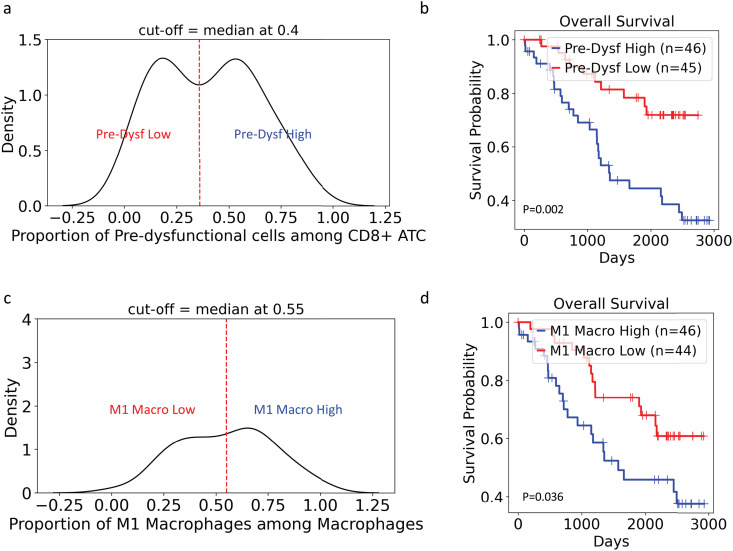
Survival analyses revealed associations between proportions of pre-dysfunctional cells and M1 macrophages with OS. (A) Distribution of the proportion of pre-dysfunctional CD8 + T cells within the CD8 + ATC population in the CoMMpass dataset among NDMM samples. The samples were categorized into pre-dysfunctional high and low groups using the median cut-off (median = 0.4). (B) Kaplan-Meier survival curve demonstrates the association between CD8 + ATC proportion and OS (pre-dysfunctional high median = 1353 days, pre-dysfunctional low median not met, *p* = 0.0025). (C) Distribution of the proportion of M1 macrophages within the macrophage population in the CoMMpass dataset among NDMM samples. The samples were further categorized into M1 macrophage high and low groups using the median cut-off (median = 0.55). (D) Kaplan-Meier survival curve demonstrate the association between the proportion of M1 macrophages and OS (M1 high median = 1574 days, M1 low median not met, *p* = 0.036). ATC, activated T cells; NDMM, newly diagnosed multiple myeloma; OS, overall survival.

CoMMpass patients were classified according to their proportion of pre-dysfunctional CD8 + ATCs. Distribution of pre-dysfunctional cells was generated, and patients were grouped into pre-dysfunctional-low and -high groups using a median cut-off value of 0.4 (Fig 7A). Patients with high proportions of pre-dysfunctional cells (median = 1353 days) were significantly (*p* = 0.0025) negatively associated with OS compared to patients with low proportions of pre-dysfunctional cells (median not met) (Fig 7B). Similarly, patients were grouped into M1 macrophage-low and -high groups using a median cut-off value of 0.55 (Fig 7C). Patients with a high proportion of M1 macrophages (median = 1574 days) had significantly (*p* = 0.036) worse OS compared with patients with low proportions of M1 macrophages (median not met) (Fig 7D). For both pre-dysfunctional CD8 + ATC and macrophage populations, no association with PFS was observed (S10B and S11 Figs). Finally, no association was observed between activated cDC proportions and survival (S10A Fig).

### Ligand-receptor interaction modelling demonstrates dynamic interplay between the tumor and microenvironment

**Fig 8:**
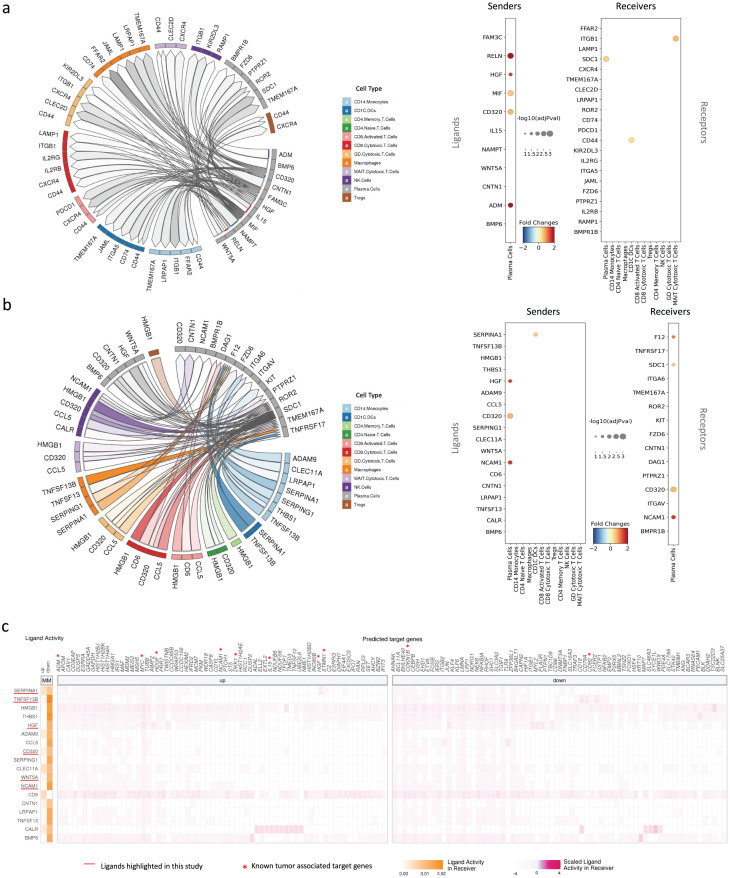
Ligand-receptor interaction modelling revealed potential interplay between tumoral plasma cells and immune populations. (A) The circos plot (left) shows the interactions when plasma cells are selected as senders and the immune cells as receivers. The dot plot (right) shows significantly differentially expressed ligands/receptors in sender/receiver cells (*p* < 0.05). (B) The circos plot (left) shows the interactions when the immune cells are selected as senders and plasma cells as receivers. The dot plot (right) shows significantly differentially expressed ligands/receptors in sender/receiver cells (*p* < 0.05). (C) The activity of the ligands from immune cells and their regulatory potential over significantly upregulated and downregulated genes in plasma cells are shown. The order of the ligands (top to bottom) represents their rank on the prioritization. The highlighted ligands either had significant differential expression in the cell types of interest or were observed in our population shift study. All the plots in this figure are obtained using the results from the differential expression analysis of MM versus premalignant stages (MGUS & SMM) contrast. DC, dendritic cell; MGUS, monoclonal gammopathy of unknown significance; MM, multiple myeloma; NK, natural killer; SMM, smoldering MM; Treg, regulatory T cell.

First, the effect of the plasma cells on their surrounding immune cells was evaluated (Fig 8A). Among the top 50 interactions, 11 plasma cell ligands interacting with 21 receptors from various immune cell types were identified (S6 File). Only ITGB1 in MAIT cytotoxic T cells and CD44 in CD1C + DCs were differentially expressed upon disease progression in the immune microenvironment. Most of the plasma cell ligands showed upregulation in patients with MM, including RELN, HGF, MIF, CD320 and ADM. Among these ligands, MIF, CD320 and HGF have potential interactions with many immune cell types via multiple receptors. Additional interesting ligands included FAM3C, ADM and IL15. These ligands, although not identified as significantly upregulated, can be found in a secreted form.

The potential for immune cells in the microenvironment to communicate differentially with the plasma cells upon MM progression was also investigated (Fig 8B). A total of 18 immune cell ligands interacting with 15 plasma cell receptors were identified (S7 File). Among the identified receptors, F12, CD138 (SDC1) and B-cell maturation antigen (TNFRSF17) were observed to interact with multiple ligands from multiple immune cell types. Many other ligands that were upregulated within the plasma cells, including HGF, CD320, NCAM1 and ADM, were also observed.

Ligands and their potential downstream effects in plasma cells were further investigated. Interestingly, BAFF (TNFSF13B), for which expression was found to be associated with population shifts in macrophages, was amongst the top predicted ligands with potential to alter the expression of genes that are differentially expressed between MM cells and plasma cells in the premalignant stages. In this context, 2 key myeloma markers, MYC and DKK1, as well as many other cancer-associated genes, including ADM, HDGF, IL15, HGF, STMN1, CDKN1B and CD82, were potentially regulated by the predicted ligands (Fig 8C). Particularly, ADM, MYC and CDKN1B were predicted to be regulated by numerous ligands. It is noteworthy that these observations are exploratory only and important ligand receptor pairs will need further validation. However, predicted ligand-receptor interaction cascades shed light on complete succession of events potentially involved in or arriving as a consequence of progression from premalignant stages to MM.

## Discussion

Despite recent treatment advancements, MM remains an incurable disease. Comprehensive and deep molecular profiling of the tumor plasma cells and surrounding immune populations advance our understanding of disease progression and can inform the identification of novel therapeutic targets. In this study, a single-cell transcriptomic atlas comprising >120 subjects from 4 cohorts, including healthy volunteers and patients with MGUS, SMM and MM, was generated. A multi-modal single-cell omics approach was applied to investigate the molecular changes that take place in tumoral plasma cells and immune populations present in the tumor microenvironment during disease progression.

Identification of immune populations from single-cell multi-omics data in the BM is a challenging task. Cells can be identified into different subgroups based on phenotypic and/or functional characteristics. Phenotypic cell surface markers and their abundance are key determinants for an accurate classification. On the other hand, functional markers can provide a deep understanding of the current functional state of the cells. In this study, both transcriptomics and proteomics were combined to optimize the identification of distinct cell types.

In the plasma cells collected from healthy volunteers and patients with MGUS, SMM, and MM, both transcriptional similarity and heterogeneity were observed among patients of the same disease stage. Although transcriptional subclonality was observed for some subjects, within-patient plasma cell transcriptomes were found to be highly similar. As in previous studies, DE analysis confirmed the altered expression of MM marker genes, including FRZB, DKK1, MYC, CCND1 and downregulation of some tumor suppressor genes, including CD81 and CD99. Interestingly, analysis of signaling pathways revealed that despite inter-patient heterogeneity, some differentially expressed pathways, such as MYC signaling, initiated early at premalignant stages, while others, such as oxidative phosphorylation, were observed only in MM. Other pathways, such as WNT signaling and NOTCH signaling, were subject-specific and observed at all stages.

DE analysis of cells in the tumor microenvironment revealed activation of inflammatory pathways increasing from healthy volunteers to MM. For example, activation of TNFα was observed in many cell types at MGUS, indicating that the initial immune cell responses may be triggered by TNF release, followed by IFN release as the disease becomes symptomatic. Moreover, among the immune-related pathways enriched in the immune microenvironment, only IFNα was observed to be activated by the plasma cells in MM. In future studies, it may be of interest to investigate whether activation of IFN pathways is a driver, or a consequence, of disease progression. Although IFNα was investigated as a therapeutic molecule in early myeloma treatment trials [41], it was shown that co-cultures with IFNα-producing plasmacytoid DCs demonstrated higher plasma and MM cell proliferation [42,43].

Among the different inflammatory and immune subpopulations, significant population shifts were observed in CD8 + T cells, DCs and macrophages, and each of these observations was further validated using the set of independent validation datasets. In CD8 + T cells, there was a gradual population shift towards a highly active state, where these cells also exhibited gene expression consistent with signals of dysfunctionality. This pre-dysfunctional T-cell population was also recently described by Yan et al. and Leun et al., who investigated transcriptional changes during CD8 + T-cell differentiation [33,44]. Leun et al. examined data from various cancers and proposed an elegant differentiation model for intra-tumoral CD8 + T cells. The authors described a differentiation trajectory from a naive-like state towards GZMK+ pre-dysfunctional and cytotoxic states, where the cells in these 2 states can interchange. Pre-dysfunctional T cells acquire additional markers of dysfunctionality and finally transform into defective T cells. The current study suggests that this activation trajectory also occurs in MM, confirming the findings of previous reports on T-cell dysfunctionality [45–48]. In addition, this study also identified that CoMMpass patients with high proportions of pre-dysfunctional T cells had inferior OS outcomes (median of ~3.7 years) suggesting that T-cell dysfunctionality may be prognostic given that median OS estimates for the entire CoMMpass cohort exceeds 6 years [9]. Pilcher et al. have recently shown GZMK+ CD8 + exhausted T cells to be associated with rapid progression in patients with MM [49]. Finally, recent studies have suggested that immune status may be associated with clinical response to therapy [50,51] and thus, identification of T cell populations with a pre-dysfunctional phenotype may have important implications for therapy in newly diagnosed multiple myeloma.

In addition to the changes in the T-cell compartment upon disease progression, alterations in macrophages were observed. Tumor-associated macrophages have been shown to play a pivotal role in MM, contributing to proliferation, angiogenesis, immunosuppression, and drug resistance [52,53]. In this study, there was a population shift towards a highly pro-inflammatory M1 population with increased BAFF expression, and evidence that this shift was initiated at MGUS and SMM stages. Further, a high proportion of these cells was associated with poor OS in CoMMpass patients. A potential mechanism for such a negative effect is explained by BAFF expression. BAFF is a known inducer of proliferation and survival in MM cells [54–56]. The ligand-receptor interaction analysis in this study prioritized BAFF as 1 of the top-ranked ligands that could contribute to dysregulation of genes along the transformation from premalignant stages to MM. Although M1 macrophages are considered to be pro-inflammatory or anti-tumoral, and M2 macrophages are considered to be anti-inflammatory or pro-tumoral in cancer research [57], BAFF-expressing M1 macrophages have been found to be associated with bortezomib resistance [58,59], which is an important consideration as bortezomib is often incorporated into first-line therapy. Considering the further role of macrophages in bone formation [60,61], the role of macrophages in MM progression may be broader than a pro- or anti-tumoral categorization. Overall, additional studies are needed to understand the impact of macrophages on MM.

Similar to previous studies, a proportional decrease in cDCs in BM samples of patients with MM was observed [62,63]. Although cDCs in the MM microenvironment are thought to be functionally deficient [62–66], some studies have shown that these cells have the capacity to activate CD8 + T cells and to promote MM tumor progression in vitro [67–69]. Although the proportion of cDCs decreased during disease progression, there was a shift towards an active and mature phenotype, with production of pro-tumorigenic molecules, including IL1B, CXCL16, VEGFA and HBEGF.

Finally, potential cell-cell interactions were evaluated to investigate their role in disease progression from the premalignant stages to active MM. DE analysis, followed by cell-cell interaction analysis, was used to predict which ligands and receptors the plasma cells may be interacting with in the tumor microenvironment, and vice versa. This study highlights the potential effect of MIF, IL15, CD320, HGF and FAM3C on the shift in immune populations during the premalignant stage transition to active MM. The interaction of MIF with its receptors (CD44, CD74, CXCR4) are known drivers in late stage MM and other cancers [70,71] and are involved in a variety of mechanisms, including homing to BM [72], pro-tumoral M0 macrophage differentiation [73] and resistance to therapy [74,75]. Likewise, IL15 [76–78] and HGF [79–81] are known regulators of the inflammatory response in cancer. Although CD320 is known to be associated with B-cell survival and proliferation [82,83], and FAM3C is a known inducer of osteoblast differentiation [84,85], their effect on the tumor microenvironment and their role in MM progression is subject to further research. Concerning the impact of the immune compartment on MM progression, SERPINA1 and BAFF (TNFSF13B) were identified as ligands that may play a role in MM development. It is well known that BAFF has the potential to enhance growth and survival of plasma cells; however, the role of SERPINA1 in MM requires further investigation. In addition to immune populations, potential interactions between plasma cells that can influence MM progression were also identified. These interactions possibly generate a positive feedback loop in which the expression of signaling molecules, such as HGF, CD320 and WNT5A, induces expression of MYC, STMN1 and CD82, while an increase in adhesion molecules, such as MIF and NCAM1, facilitates these interactions by enabling proximity between the malignant cells. An increase in intercellular adhesion may also cause bone lesions [86,87], increase cellular mobility and contribute to resistance to therapy [88]. Although many ligand receptor relationships mentioned herein require additional validation beyond the scope of this manuscript, this analysis provides insights into potential interactions that exist between the tumor and cells in the tumor microenvironment during disease progression.

**Fig 9:**
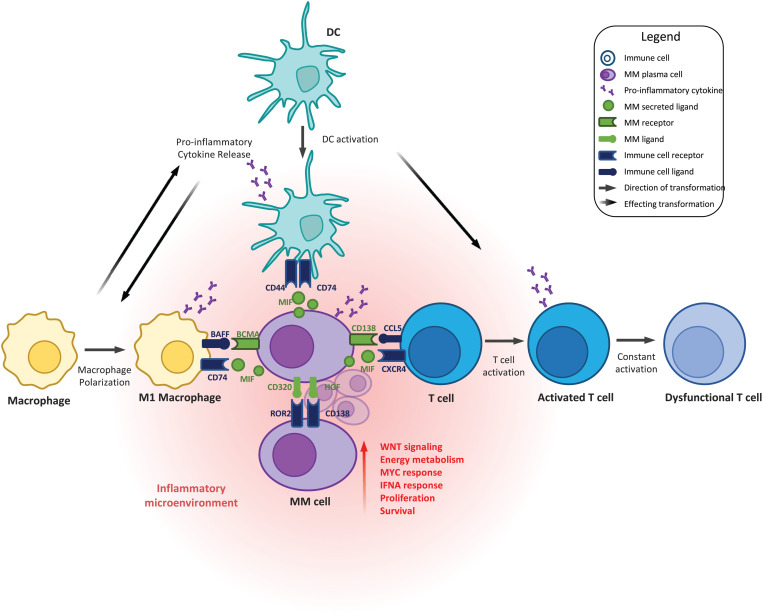
Proposed model of MM progression with dynamic interplay between malignant plasma cells and immune populations. Each cell type is represented with a different color: MM, purple; DCs, turquoise; macrophages, yellow, T cells, blue. The MM plasma cell ligands and receptors are represented in green. Ligands and receptors from the immune populations are represented in dark blue. The straight arrows show the direction of the transformation. The arrows with descending color represent the direction of the effect on transformation. The orange cloud represents the inflammatory environment. DC, dendritic cell; MM, multiple myeloma.

In conclusion, in this study, an atlas of the BM microenvironment was generated and used to investigate disease drivers from premalignant stages of MGUS and SMM to MM. An increased inflammatory response was observed from MGUS to SMM to MM underscoring that inflammation plays a key role in the progression of MM from premalignant stages. Changes in immune cell population phenotypes (e.g., macrophages, CD1C + DCs, and CD8 + ATCs) and a dynamic interplay between malignant plasma cells and these immune populations through ligand/receptor interactions, such as B-cell maturation antigen/BAFF or MIF/CD44/CD74, takes place during progression to MM (Fig 9). Higher levels of pre-dysfunctional CD8 + ATCs at MM diagnosis was associated with inferior OS, however the impact of these cell populations on therapy response in NDMM patients remains to be investigated. Overall, this study suggests that early intervention to modulate the inflammatory response in premalignant stages of the disease may help to limit disease complexity and progression to MM.

## Materials and methods

### Ethics approval and consent to participate

The study protocol was reviewed and approved by the leading ethics committee from the coordinating site, Comité d’Ethique Hospitalo-Facultaire Universitaire de Liège, and by local ethic committees of the participating sites including EC UZ Gent, EC UZ Leuven, EC Clinique Universitaires de Mont Godinne, EC Institut Jules Bordet, EC AZ Sint-Jan Brugge-Oostende. All patients provided written informed consent.

### Sample collection

BM aspirates were collected at 6 Belgian centers from subjects across 4 cohorts: 31 healthy volunteers, 28 patients with MGUS, 32 patients with SMM and 32 patients with newly diagnosed MM (Fig 1A). MM was defined using the updated criteria for the diagnosis of MM by the International Myeloma Working Group (see S1 Appendix for additional details) [89]. BM aspirates were diluted at a ratio of 7:1 with phosphate-buffered saline (PBS) and filtered through a 100 µm filter. BM mononuclear cell layers were isolated after Ficoll-Paque PLUS (density 1.077 + /– 0.001 g/mL) gradient separation, washed 3 times with PBS and stored in liquid nitrogen at 5–8 × 10^6^ cells/ml in freezing medium (90% fetal bovine serum + 10% dimethyl sulfoxide).

### Single-cell sequencing

Samples were thawed at 37ºC and diluted in a prewarmed RPMI-1640 medium before centrifugation. Cell pellets were resuspended in cold PBS + 1% bovine serum albumin. For each sample, 0.2 × 10^6^ cells were stained using a tagged-antibody pool (see S1 Appendix for additional details) following the manufacturer’s procedure (BioLegend, San Diego, California, USA). After staining, cells were resuspended in PBS + 0.04% bovine serum albumin at a concentration of 1000 cells/µl, filtered using a FACS tube with a cell strainer cap and processed immediately according to the 10x Genomics Chromium Single Cell V(D)J with Feature Barcoding protocol. Viability was assessed by Moxi Flow Flow Cytometer using propidium iodide, where a median of 89.2%, a minimum of 60.4% and a maximum of 99.4% was observed. Twenty thousand cells were loaded onto 10x chips. For each chip, 1 sample from each cohort was processed and loaded in a randomized order. Single-cell multi-omics profiling, including RNA, BCR and ADT 10x sequencing, was conducted according to the 10x Genomics manufacturer instructions (10x Genomics, CG000186 Rev C). Library pools were sequenced using an Illumina NovaSeq 6000 following 10x Genomics’ instructions to reach a median sample sequencing saturation of 90.7% for the whole dataset (lowest sample saturation observed was 63.5%).

### Single-cell multi-omics data analysis

Sample demultiplexing, barcode processing, alignment to the human transcriptome (GRCh38), single-cell 5′ gene, V(D)J and feature barcode counting were performed using Cell Ranger Single-Cell Software Suite (Cell Ranger-4.0.0, 10x Genomics). Each data type (scRNAseq, ADT-seq, BCRseq) was then independently preprocessed per sample and was subsequently concatenated into a single dataset for further downstream analysis. Unless stated otherwise, for each data analysis step, related functions from the Scanpy [90] (version 1.6.1) Python package were used with default parameters.

### scRNA-seq data preprocessing

Empty droplets and dying cells were filtered out based on unique molecular identifier counts <650, minimum number of genes <300, and mitochondrial gene expression $\begin{array}{c} \;> \end{array}$20%. Doublet detection was conducted by Scrublet (version 0.2.1) [91]. Droplets with a Scrublet doublet score >0.3 and genes expressed in <3 cells were excluded. Following the initial filtering, a count per million normalization was applied with a scale factor of 10^4^ and the data were log transformed. Highly variable genes were detected with ‘Seurat’ flavor [92]. For the downstream analyses, immunoglobulin genes were removed from the dataset. Cell cycle scores were calculated using *score_genes_cell_cycle* function from Scanpy using the gene sets identified by Kowalczyk et al. [93].

### Single-cell ADT sequencing data preprocessing

Droplets with unique molecular identifier counts <500 were excluded. The data were normalized using centered log-ratio transformation [94].

#### scBCR-seq data preprocessing.

Cell Ranger-filtered BCR-seq output contig files from individual samples were concatenated into a single file. Using the Scirpy (version 0.6.1) library [95], with parameters *receptor_arms = all* and *dual_ir = primary only*, observed clonotypes were defined based on CDR3 nucleic acid sequences. To assess clonal expansion, per-sample clonotype definitions were generated using both CDR3 nucleic acid sequences and sample identifiers. Per-sample clonotypes observed in ≥10 cells were considered expanded malignant clonotypes.

#### Dataset integration and visualization.

ADT-seq and scRNAseq data were integrated independently for all samples. For both ADT-seq and RNA integrated datasets, principal component analysis and Harmony (version 1.0) batch correction [95], using theta = 0, was performed. Nearest neighborhood networks were constructed using a cosine distance. Each integrated dataset was projected onto 2-dimensional space applying the UMAP [96] algorithm based on the standard settings of Scanpy. The data were grouped by Leiden clustering [97] within Scanpy via multiple resolutions.

#### Cell typing.

To save time and computational resources, a reference dataset for automated cell typing was generated running the data analysis pipeline on an initial set of 12 samples comprising 3 subjects from each cohort. The cell types were identified in 3 annotation steps using the expression of protein and gene markers as presented in Fig 1B and S8 File (see also S12A Fig). See S1 Appendix for additional details.

#### Proportional comparison analysis.

Proportional changes among cohorts were assessed using the Mann–Whitney U test, comparing data from patients with MGUS, SMM and MM with data from healthy volunteers. Plasma cells were excluded from this analysis; changes were considered significant at a threshold of *p* < 0.05.

#### Differential expression analysis.

Two types of differential expression (DE) methodologies were applied. For contrasts involving comparisons at the subject level, a pseudobulk approach using the muscat [98] (version 1.4.0) R package was used (see S1 Appendix for additional details). To compare cell clusters, Wilcoxon rank sum test was applied. Significantly down- and upregulated genes were identified based on adjusted *p* value (padj < 0.05).

#### Gene set scoring and enrichment analyses.

Hallmark and biological process gene sets were obtained from the MsigDB website [99], and gene set scores were calculated using the *scanpy score_genes* function with normalized counts. Gene set enrichment analysis was conducted using the R package fgsea [100] (version 1.12.0) with *nperm = 10,000*, using the test score obtained from the Wilcoxon rank sum test as the ranking variable. Due to high variation in the number of genes among the gene sets, a minimum of 30 genes and a maximum of 200 genes for the analysis of biological process gene sets was required.

#### Ligand-receptor interaction modelling.

To better understand the molecular mechanisms and changes in the cell-cell interactions that take place during the transformation of premalignant to malignant cells, MultiNicheNet [101] (version 1.0.0) was used following the *detailed_analysis_steps_MISC.md* vignette provided by the developers, with the differential expression results from the muscat differential gene expression analysis for the contrast MM versus premalignant ([MGUS+SMM]/2). The top 12 cell types that had the most significantly differentially expressed genes between MGUS and SMM versus MM were analyzed (S9 File). This method allows the modeling and prioritization of potential ligand-receptor interactions between cell types by evaluating known ligand-receptor interactions from public databases, along with their gene expression abundance and DE of the downstream genes in the contrast groups. The outcome of both directions of tumoral immune-cell and immune tumoral-cell interactions were investigated.

#### Survival regression analysis.

Survival data were evaluated using the Kaplan-Meier method. A Cox proportional hazards model was used to assess the association of cell-type abundance with progression-free survival (PFS) and overall survival (OS). The analyses were conducted using the lifelines Python library (version 0.27.4).

#### Validation studies using external datasets.

Three external datasets (Zavidij et al. [15], de Jong et al. [17], and the CoMMpass study [9,102] were used to validate our findings. See S1 Appendix for additional details on the study data used. Data from all 3 studies were run through the same pipeline used to analyze the data, and the cell types of interest were identified using Leiden clustering. To accommodate the lack of cell surface protein information in the external datasets, manual gating (CD3E+ or CD3D + ; CD4–; and CD8A+ or CD8B+) was applied for the identification of CD8 + T cells.

#### CyTOF analysis.

BM samples from HV, MGUS, SMM, and MM patients were processed and run on a Helios system. Manual gating was performed using Cytobank and batch effect correction was performed using cyCombine (v0.2.1.5). Unsupervised clustering was performed using FlowSOM. Clusters were grouped into metaclusters, and marker expression in each metacluster was manually reviewed to identify known immune cell subsets and subpopulations. Prior to statistical abundance analysis, plasma cells, granulocytes, and cells with unclear annotations were removed. Differences in population sizes were assessed, and multiple testing correction was applied. See S1 Appendix for additional details.

## Supporting information

S1 Appendix Supplementary methods. (DOCX)

S1 Fig Frequency of cell populations in HV, MGUS, SMM, and MM by CyTOF. Populations include CD19 + CD27- B cells (A), CD14 + CD16- monocytes (B), CD3 + CD8 + CD45RO+CD27 + naive CD8 + T cells (C) and CD3 + CD8 + CD45RO+PD-1 + TIGIT+ predysfunctional CD8 + T cells (D).
(EPS)

S2 Fig Frequency of NK cell subpopulations in HV, MGUS, SMM, and MM by CyTOF. Subpopulations include CD56dimCD16- NK cells (A), CD56dimCD16 + CD69 + Activated NK Cells, CD56dimCD16 + CD57 + Mature NK cells (C) and CD56brightCD27 + NK Cells.
(EPS)

S3 Fig Top 10 up- and down-regulated hallmark gene sets (A). Top 10 up- and downregulated BP gene sets (B). For both subsections, the GSEA analyses are conducted contrasting pre-dysfunctional CD8 + ATCs with other CD8 + ATCs. ATC, activated T cell; BP, biological process; GSEA, gene set enrichment analysis; NES, normalized enrichment score.
(EPS)

S4 Fig GSEA results of M1 macrophages. Top 10 upregulated hallmark gene sets. No significantly downregulated gene sets have been identified (A). Top 10 up- and downregulated BP gene sets (B). For both subsections, the GSEA analyses are conducted contrasting M1 macrophages with other macrophages. BP, biological process; GSEA, gene set enrichment analysis; NES, normalized enrichment score.
(EPS)

S5 Fig Macrophage, CD1C + DC and CD14 + DC populations. Proportional differences among cohorts for M2 macrophages (A). Each dot represents an individual subject. The proportional differences between each disease state versus HVs are compared and the significance was calculated using the Mann–Whitney U test: *p < 0.05, **p ≤ 0.005, ***p ≤ 0.0005. Expression profiles of proliferation markers define the proliferating cell group (B). Investigation of CD14 + DCs (C). UMAP of CD14 + DCs, colored by the obtained clusters, the proportional difference and density plots among subject cohorts show transcriptional shifts (C top and center). Highlighted area represents the region where the cells in cluster 1 reside. Expression profile of the markers defining active CD1C + DC subgroup (C right). The figure colors range from minimum value (yellow) to the 99^th^ percentile of the value distribution (red). Gene set scores of inflammatory pathways highlight inflammation responsive regions on the CD14 + DCs UMAP (C bottom). The figure colors range from minimum value (blue) to the 99^th^ percentile of the value distribution (red). DC, dendritic cell; HV, healthy volunteer; IFN, interferon; MGUS, monoclonal gammopathy of undetermined significance; MM, multiple myeloma; SMM, smoldering MM; TNF, tumor necrosis factor.
(EPS)

S6 Fig GSEA results of active CD1C + DCs. Top 10 up- and downregulated hallmark gene sets (A). Top 10 up- and downregulated BP gene sets (B). For both subsections, the GSEA analyses are conducted contrasting active CD1C + DCs with other CD1C + DCs. BP, biological process; DC, dendritic cell; GSEA, gene set enrichment analysis; NES, normalized enrichment score.
(EPS)

S7 Fig GSEA results of cluster1 of CD14 + DCs. Top 10 upregulated hallmark gene sets. No significantly downregulated gene sets have been identified (A). Top 10 up- and downregulated BP gene sets (B). For both subsections, the GSEA analyses are conducted contrasting cluster 1 of CD14 + DCs with other CD14 + DCs. BP, biological process; DC, dendritic cell; GSEA, gene set enrichment analysis; NES, normalized enrichment score.
(EPS)

S8 Fig Validation study of de Jong et al. Findings on cDCs (CD1C + and CD14 + DCs) (A). UMAP of cDCs, colored by the obtained clusters and the dense regions per cohort (A top left). Expression profile of active CD1C + DC markers within cDC clusters (A top right). The figure colors range from minimum value (yellow) to the 99^th^ percentile of the value distribution (red). Gene set scores of IFN and TNF pathways demonstrate inflammation-associated regions (A bottom left). The figure colors range from minimum value (blue) to the 99^th^ percentile of the value distribution (red). Proportional differences among cohorts for CD14 + DC clusters (A bottom right). Each dot represents an individual subject. The proportional differences between each disease state versus HVs are compared and the significance was calculated using the Mann–Whitney U test: *p < 0.05, **p ≤ 0.005, ***p ≤ 0.0005. Findings on CD8 ATCs (B). Two clusters are identified. Between HVs and MM subjects, the dense regions differ (B top left). Expression profile of CD8 + ATC markers within CD8 ATC clusters (B right). The figure colors range from minimum value (yellow) to the 99^th^ percentile of the value distribution (red). Gene set score of TNFα pathway demonstrates inflammation-associated regions (B left). The figure colors range from minimum value (blue) to the 99^th^ percentile of the value distribution (red). Proportional differences among cohorts for CD8 + ATC clusters (B bottom center). Each dot represents an individual subject. The proportional differences between each disease state versus HVs are compared and the significance was calculated using the Mann–Whitney U test: *p < 0.05, **p ≤ 0.005, ***p ≤ 0.0005. ATC, activated T cells; cDC, classical DC; DC, dendritic cell; HV, healthy volunteer; IFN, interferon; MM, multiple myeloma; TNF, tumor necrosis factor.
(EPS)

S9 Fig Validation study of Zavidij et al. Findings on macrophage population. UMAP of cDCs, colored by subgroups as well as the dense regions per cohort (top). Gene set scores of inflammatory pathways highlight inflammation-responsive regions on the macrophage UMAP (8 center). The figure colors range from minimum value (blue) to the 99^th^ percentile of the value distribution (red). Distribution of BAFF expression (bottom left). The cells are categorized by the local minimum at 0.68 into BAFF-high and BAFF-low categories. Density UMAP highlights the dense regions of each group on the translation landscape of macrophages. Proportional differences among cohorts for macrophage subtypes (right center). Each dot represents an individual subject. The proportional differences between each disease state versus HVs are compared and the significance was calculated using the Mann–Whitney U test: *p < 0.05, **p ≤ 0.005, ***p ≤ 0.0005. Expression profiles of marker genes that define macrophage subpopulations (8 bottom right). cDC, classical dendritic cell; HV, healthy volunteer; IFN, interferon; MGUS, monoclonal gammopathy of undetermined significance; MM, multiple myeloma; SMM, smoldering MM; TNF, tumor necrosis factor.
(EPS)

S10 Fig Validation study of CoMMpass dataset. Our findings on cDCs (CD1C + and CD14 + DCs) (A). UMAP of cDCs, colored by identified clusters as well as the gene set scores of inflammatory response and TNFα signaling (A top left). The figure colors range from minimum value (blue) to the 99^th^ percentile of the value distribution (red). Expression profile of marker genes, used to identify active and inactive CD1C + DCs, within cDC clusters (A top right). The subjects are categorized based on the distribution of proportion of cells in cluster 1 (cDC C1) (A bottom). The median value of 0.67 is used as a cut-off to group subjects into cDC C1 high and low categories. Survival analyses for CoMMpass patients with high and low proportions of cDC C1 populations showed no difference in PFS (C1 high median = 1094 days, C1 low median = 869 days, *p* = 0.25) or OS (C1 high median = 2444 days, C1 low median not met; *p* = 0.32). Our findings on CD8 + ATCs (B). UMAP representation of the CD8 + ATCs colored by CD8 + ATC subgroups and TNFα signaling (B top). The figure colors range from minimum value (blue) to the 99^th^ percentile of the value distribution (red). No difference in PFS outcome was observed in CoMMpass patients with high and low proportions of pre-dysfunctional CD8 + ATCs (pre-dysfunctional high median = 778 days, pre-dysfunctional low median = 1010 days; *p* = 0.29) (B bottom). ATC, activated T cell; cDC, classical DC; DC, dendritic cell; OS, overall survival; PFS, progression-free survival; TNF, tumor necrosis factor.
(EPS)

S11 Fig Validation study of CoMMpass dataset: Findings on macrophages. UMAP representation of the macrophages colored by macrophage subgroups and the expression profile of marker genes define each macrophage subgroup (top). UMAP representation of the macrophages colored IFNα and TNFα signaling (right). The figure colors range from minimum value (blue) to the 99^th^ percentile of the value distribution (red). The subjects are categorized based on the distribution of proportion of cells in M1 macrophages (center). The local minimum at 0.38 is used as a cut off to group subjects into M1-high and -low categories. No difference in PFS outcome was observed in CoMMpass patients with high and low proportions of M1 macrophages (M1 high median = 772 days, M1 low median = 1010; *p* = 0.17). Distribution of BAFF expression (bottom left). The cells are categorized by the local minimum at 0.68 into BAFF-high and BAFF-low categories. IFN, interferon; PFS, progression-free survival; TNF, tumor necrosis factor.
(EPS)

S12 Fig Reference based automated cell typing strategy. Reference data generation steps, visualized by ADT and RNA UMAPs using the colors of cell types at different annotation levels (A). The data was generated using 12 initial samples, where each cohort was represented with 3 samples. Reference data generation steps are demonstrated (B top). The integration step of automated cell typing strategy into the data processing pipeline is demonstrated (B bottom). Light red, reference RNA data; dark red, query RNA data; light green, reference ADT data; dark green, query ADT data; light yellow, integrated dataset of RNA and ADT. ADT, antibody derived tag; HSPC, hematopoietic stem and progenitor cell; mDC, myeloid dendritic cell; NK, natural killer; pDC, plasmacytoid dendritic cell.
(EPS)

S1 File Differential expression results of plasma cells. Description: Each disease cohort (sheet names) is compared with HVs using muscat.
(XLSX)

S2 File Differential expression results of diseased versus non-diseased plasma cells. Description: The expression profile of diseased plasma cells was contrasted with healthy (non-diseased) plasma cells, using muscat.
(XLSX)

S3 File Differential expression results of immune cells of MGUS patients. Immune populations (sheet names) of MGUS patients are compared with immune populations of HV, using muscat.
(XLSX)

S4 File Differential expression results of immune cells of SMM patients. Immune populations (sheet names) of SMM patients are compared with immune populations of HVs, using muscat.
(XLSX)

S5 File Differential expression results of immune cells of MM patients. Immune populations (sheet names) of MM patients are compared with immune populations of HVs, using muscat.
(XLSX)

S6 File Top 50 ligand-receptor modelling results modelling the interactions from plasma cells to immune populations. MultiNicheNet analysis is conducted for modelling the interactions between the ligands from plasma cells and the receptors from the immune populations. The results from MM versus premalignant comparison are used as input for the modelling.
(XLSX)

S7 File Top 50 ligand-receptor modelling results modelling the interactions from immune populations to plasma cell. MultiNicheNet analysis is conducted for modelling the interactions between the ligands from immune populations and the receptors from the plasma cells. The results from MM versus premalignant comparison are used as input for the modelling.
(XLSX)

S8 File Gene and protein markers used for cell typing using level methodology presented in S11 Fig. Each sheet represents one level of cell typing with cell (sub)populations expected as positive or negative.
(XLSX)

S9 File Differential expression results of comparing MM patients with premalignant (MGUS and SMM) patients. The expression profile of MM cells was contrasted premalignant (MGUS and SMM) cells, using muscat.
(XLSX)
