# Supplementary material for: Immunophenotypic changes in the tumor and tumor microenvironment during progression to multiple myeloma
Source: PLoS Genet. 2025 Oct 7;21(10):e1011848. doi: 10.1371/journal.pgen.1011848 (PMC12558612; doi:10.1371/journal.pgen.1011848)
Supplement: S1 Appendix — (DOCX) [file pgen.1011848.s001.docx]

**S1 Appendix**

[Supplementary methods 2](#_Toc1074148103)

[Subject cohort categorization criteria 2](#_Toc125932330)

[Single-cell sequencing 5](#_Toc1238054575)

[Cell typing 8](#_Toc1076876987)

[Differential expression analysis 9](#_Toc1481995661)

[Validation studies using external datasets 9](#_Toc970465042)

[CyTOF Analysis 12](#_Toc355958473)

# Supplementary methods

## Subject cohort categorization criteria

All participants were aged above 18 years. Any potential participant who met any of the following criteria were excluded from participating in the study:

- History of drug or alcohol abuse.
- Female who might be pregnant or was breast-feeding at the time of enrollment.
- Active/other malignancy or history of malignancy (other than multiple myeloma [MM]) within 3 years prior to screening. Exceptions were squamous and basal cell carcinomas of the skin and carcinoma in situ of the cervix, or malignancy which in the opinion of the investigator were cured.
- Active autoimmune disorder.
- Signs of active infection, at the discretion of the investigator.
- Concomitant immune-suppressive medication including prednisone >5 mg daily.

Prospective smoldering MM or symptomatic MM participants:

- No history of or current anti-myeloma therapy.
- No prior use of bisphosphonates or denosumab.

The following criterion were used for inclusion in the specific cohorts:

- Healthy volunteer >50 years of age

Monoclonal gammopathy of unknown significance (MGUS):

- Immunoglobulin (Ig) G or A MGUS (all criteria must be met):
  - Serum monoclonal protein (IgG or IgA or IgM) <3 g/dL.
  - Clonal bone marrow (BM) plasma cells <10%.
  - No myeloma-defining events (defined below).
- OR light chain MGUS (all criteria must be met):
  - Abnormal serum free light chain ratio (<0.26 or >1.65).
  - Increased level of the appropriate involved light chain (increased κ serum free light chain in patients with ratio >1.65 and increased λ serum free light chain in patients with ratio <0.26).
  - No immunoglobulin heavy chain on immunofixation.
  - Clonal BM plasma cells <10%.
  - Urinary monoclonal protein <500 mg/24h.
  - No myeloma-defining events (defined below).

Smoldering MM:

- Serum monoclonal protein (IgG or IgA) ≥3 g/dL OR
- Urinary monoclonal protein ≥500 mg/24 h AND/OR
- Clonal BM plasma cells 10–60%
- AND no myeloma-defining events or amyloidosis (no CRAB and no SLiM as detailed below)

Symptomatic MM:

- Myeloma-defining events are evidence of end-organ damage that can be attributed to the underlying plasma cell proliferative disorder, especially:
  - C: Calcium elevation (>11 mg/dL or >1 mg/dL higher than upper limit of normal)
  - R: Renal insufficiency (creatinine clearance <40 mL/min or serum creatinine >2 mg/dL)
  - A: Anemia (hemoglobin <10 g/dL or 2g/dL < normal)
  - B: Bone disease (≥ 1 lytic lesion on skeletal radiography, computed tomography, or positron emission tomography-computed tomography). If BM had <10% clonal plasma cells, >1 bone lesion was required to distinguish from solitary plasmacytoma with minimal marrow involvement.
- OR, in the absence of CRAB, ≥1 of the following biomarkers of malignancy, referred to here as the SLiM criteria: S = ≥60% clonal BM plasma cells; Li = serum free light chain ratio involved:uninvolved ≥100; M => 1 focal lesion (≥5 mm each) detected by magnetic resonance imaging studies.

Note: Clonality should be established by showing κ/λ-light-chain restriction on flow cytometry, immunohistochemistry, or immunofluorescence. BM plasma cell percentage should preferably be estimated from a core biopsy specimen; in case of a disparity between the aspirate and core biopsy, the highest value should be used.

## Single-cell sequencing

**S1 Table. ADT sequencing pools.**

| **ADT** | **Protein** | **Gene** |
| --- | --- | --- |
| CD11b_TotalSeqC | CD11b | *ITGAM* |
| CD11c_TotalSeqC | CD11c | *ITGAX* |
| CD123_TotalSeqC | CD123 | *IL3RA* |
| CD127_TotalSeqC | CD127 | *IL7R* |
| CD137_TotalSeqC | CD137 | *TNFRSF9* |
| CD138_TotalSeqC | CD138 | *SDC1* |
| CD14_TotalSeqC | CD14 | *CD14* |
| CD15_TotalSeqC | CD15 | *FUT4* |
| CD152_TotalSeqC | CD152 | *CTLA4* |
| CD16_TotalSeqC | CD16 | *FCGR3A* |
| CD19_TotalSeqC | CD19 | *CD19* |
| CD197_TotalSeqC | CD197 | *CCR7* |
| CD20_TotalSeqC | CD20 | *MS4A1* |
| CD223_TotalSeqC | CD223 | *LAG3* |
| CD24_TotalSeqC | CD24 | *CD24* |
| CD244_TotalSeqC | CD244 | *CD244* |
| CD25_TotalSeqC | CD25 | *IL2RA* |
| CD269_TotalSeqC | CD269 | *TNFRSF17* |
| CD27_TotalSeqC | CD27 | *CD27* |
| CD270_TotalSeqC | CD270 | *TNFRSF14* |
| CD272_TotalSeqC | CD272 | *BTLA* |
| CD274_TotalSeqC | CD274 | *CD274* |
| CD278_TotalSeqC | CD278 | *ICOS* |
| CD279_TotalSeqC | CD279 | *PDCD1* |
| CD28_TotalSeqC | CD28 | *CD28* |
| CD3_TotalSeqC | CD3 | *CD3D* |
| CD3_TotalSeqC | CD3 | *CD3E* |
| CD33_TotalSeqC | CD33 | *CD33* |
| CD357_TotalSeqC | CD357 | *TNFRSF18* |
| CD366_TotalSeqC | CD366 | *HAVCR2* |
| CD38_TotalSeqC | CD38 | *CD38* |
| CD4_TotalSeqC | CD4 | *CD4* |
| CD45_TotalSeqC | CD45 | *PTPRC* |
| CD45RA_TotalSeqC | CD45RA |  |
| CD45RO_TotalSeqC | CD45RO |  |
| CD56_TotalSeqC | CD56 | *NCAM1* |
| CD66b_TotalSeqC | CD66b | *CEACAM8* |
| CD69_TotalSeqC | CD69 | *CD69* |
| CD8_TotalSeqC | CD8 | *CD8A* |
| CD8_TotalSeqC | CD8 | *CD8B* |
| CD80_TotalSeqC | CD80 | *CD80* |
| GPRC5D_TotalSeqC | GPRC5D | *GPRC5D* |
| HLA-DR_TotalSeqC | HLA-DR | *HLA-DRA* |
| HLA-DR_TotalSeqC | HLA-DR | *HLA-DRB1* |
| TIGIT_TotalSeqC | TIGIT | *TIGIT* |

ADT, antibody derived tag.

## Cell typing

The whole cell typing dataset was annotated using the generated reference dataset and an adapted version of SingleR (version 1.4.1) (1) (Fig S12B). The adapted SingleR was run on single-cell RNA sequencing (scRNA-seq) data to detect the cell types identified on the second step of reference generation, where the subtypes were identified using only scRNA-seq data. Next, for each subtype that could be further subcategorized using antibody derived tag (ADT) data, the adapted SingleR was run using ADT data for each cell type. Finally, the annotations were concatenated as a single annotation output with further Leiden clustering applied for the identified cell groups in the whole dataset. The clusters that showed distinct characteristics were considered as independent cell types and misclassifications were corrected. In cases in which certain clusters were characterized by high mitochondrial percentage and/or low number of genes within a cell population, they were labelled as low quality and removed from downstream analyses. Inconsistent cells represented 0.0007% of the dataset.

At level 1, to avoid inconsistency between scRNA-seq and ADT data, the whole dataset was annotated for major cell types using both data types independently. The cells whose annotation did not overlap between the 2 data types were marked as ‘inconsistent’ and represented 3.35% of the dataset. Because scRNA-seq data provide more insight for the cellular state due to high-feature coverage, transcriptional data were used to define the subtypes and identify the transcriptionally distinct subtypes. At level 2, each major cell type was isolated, reprocessed (HVG selection, principal component analysis generation, batch correction and UMAP generation), Leiden clustered, and annotated based on highly ranked genes. At level 3, for those subtypes that could be further subtyped based on ADT data, the same methodology was conducted using only ADT data to classify T cells based on both transcriptional markers and cell surface protein markers. For additional information, refer to S8 File.

## Differential expression analysis

Briefly, muscat applies the quasi-likelihood method from the standard edgeR (2) pipeline on sum-aggregated counts across subjects and cell types. Before aggregation, a gene-filtering step was performed at the single-cell level, excluding the Ig genes and retaining only genes with at least ≥1 count in 10 cells across all samples. The standard muscat sample filtering was further used; for each cell type tested, only subjects with ≥10 cells were used for that contrast. Cell types represented by <3 subjects after this step were not considered for differential expression testing. Finally, to avoid any genes expressed in just a few cells, a post hoc gene filtering step was applied to remove any genes from the differential expression list expressed in <10% of the cells for a given cell-type cohort combination, followed by recalculation of the false discovery rate-corrected *p* values.

## Validation studies using external datasets

To validate these observed shifts in immune populations and investigate their relationship with progression-free survival and overall survival, external datasets from similar studies, including Zavidij et al. (3), de Jong et al. (4) and the MMRF CoMMpass study (5, 6) were used (S2 Table). For the Zavidij et al. data, Cell Ranger output (mtx) files were obtained from Gene Expression Omnibus (GEO, GSE124310). This dataset included 25,142 cells from 32 subjects, including 9 healthy volunteers, 5 patients with MGUS, 11 patients with smoldering MM and 7 patients with MM. For data from the de Jong et al. study, the fastq files were obtained from ArrayExpress (E-MTAB-9139). A total of 346,673 cells from 25 subjects composed of 12 healthy volunteers and 13 patients with MM were analyzed. For the CoMMpass study data, raw immune cell scRNA-seq data (IA-001) of patients from the CoMMpass study (NCT01454297), generated by the Multiple Myeloma Research Foundation Immune Atlas network, were obtained. Survival analysis was conducted using the ttcos, ttcpfs, censos and censpfs fields using the clinical data from the IA20 release. Only newly diagnosed MM samples were analyzed (516,394 cells from 92 patients).

**S2 Table. Summary of the validation study using external dataset.**

|  | **Zavidij et al. 2020** | **de Jong et al. 2021** | **CoMMpass cell type** | **CoMMpass survival (OS)** | **CoMMpass survival (PFS)** |
| --- | --- | --- | --- | --- | --- |
| **DCs** | NA^a^ | ✓ | ✓ | X | X |
| **Macrophages** | ✓ | NA^b^ | ✓ | ✓ | X |
| **CD8 ATC** | X | ✓ | ✓ | ✓ | X |
| Supported or validated cell types are marked with a check mark (✓), while unsupported cell types are marked with an X.  ^a^Compared with the current dataset and the de Jong et al. dataset, the Zavidij et al. dataset included a smaller number of cells. This resulted in an insufficient number of DCs for further subtyping. The main CD8+ ATC population was identified but similar subpopulations were not detected.  ^b^In the de Jong et al. dataset, macrophages were not retained due to the sample processing methodology.  ATC, activated T cell; DC, dendritic cell; NA, not available; PFS, progression-free survival; OS, overall survival. | | | | | |

## CyTOF Analysis

BM aspirates were fixed in SmartTubes for 10 minutes at room temperature according to the manufacturer’s protocol (Smart Tube, Inc., Palo Alto, CA, US) and stored at −80°C until analysis. Purified metal-conjugated antibodies were either obtained from Standard BioTools (Standard BioTools Inc) or labeled in-house using the Maxpar Antibody Labeling Kit (Standard BioTools Inc) according to the manufacturer’s protocol.

Samples were thawed in a 10°C water bath for 20 minutes, and red blood cells were lysed using a hypotonic thaw-lyse buffer (SmartTube) following the manufacturer’s protocol. If red blood cells were not completely lysed after 2 lysis steps, an additional lysis step using lyse buffer 2 (SmartTube Inc., Palo Alto, CA, US) was performed. Samples still containing red blood cells after 3 lysis steps were further purified by CD235 depletion (MACSxpress Erythrocyte Depletion Kit; Miltenyi Biotec).

Cells were washed with 25 mL PBS (Thermo-Fisher Inc, Waltham, MA, US) and with 10 mL stain buffer (BD Biosciences, San Jose, CA, US). Cells were resuspended in 1.8 mL staining buffer, counted, and divided over two tubes with a maximum of 2 million cells/tube in 2 mL staining buffer after which the surface Fc receptors were blocked by addition of 10 µL Human TruStain FcX (BioLegend, San Diego, CA, US) and incubation for 15 minutes at 4°C. Cells were incubated with a antibodies (S3 Table) for 45 min at 4°C. Cells were then washed twice in staining buffer and permeabilized by addition of 1 mL ice cold PermWash buffer (BD Biosciences) while vortexing and incubation at 4°C for 20 minutes. Cells were pelleted by centrifugation for 6 minutes at 600 g. After removal of supernatant, antibodies for intracellular staining (S3 Table) were added, and cells were incubated for 45 minutes at 4°C. Cells were washed twice in 2 mL PermWash buffer and once in 2 mL staining buffer before addition of 1 mL ice cold PermIII buffer (BD Biosciences) while vortexing and incubation at 4°C for 20 minutes. Cells were washed twice with 2 mL staining buffer, after which 80 µl 191- and 193-Iridium labeled nucleic acid intercalator (Cell-ID intercalator, Standard BioTools; dilution 1/8000 in PBS) was added, and cells were barcoded by addition of 10 µl of barcode (Cell-ID 20-plex Pd Barcoding Kit (Standard BioTools) and 100 µLof PBS and incubation for 30 minutes at 4°C. After 2 washing steps with 2 mL staining buffer and an additional wash with ultrapure water, labeled cells were dissolved in ultrapure water with EQ Four Element Calibration Beads at 3.3x10E+04 beads/mL (Standard BioTools). Barcoded samples were pooled before acquisition. Samples were acquired on a Helios system at an acquisition rate of 300 to 500 cells/minute in experiments using a maximum of 20 barcoded samples per experiment. Each experiment included extra aliquots of non-barcoded whole blood samples and cell lines for quality control purposes***.***

**S3 Table. Antibody T panel used for the CyTOF analysis of bone marrow samples.**

| **Specificity** | **Metal isotope** | **Purpose** | **Type** |
| --- | --- | --- | --- |
| Activecasp3 | 198Pt | Apoptotic cells | functional |
| CD11a | 112Cd |  | functional |
| CD137 | 158Gd | Activated NK cells | functional |
| CD152 | 156Gd | Regulatory T cells, T-cell activation | functional |
| CD160 | 159Tb |  | functional |
| CD184 | 144Nd |  | functional |
| CD223 | 173Yb | T cell co-inhibitory receptor | functional |
| CD244 | 141Pr |  | functional |
| CD269 | 166Er | Plasma cells | functional |
| CD270 | 168Er |  | functional |
| CD274 | 175Lu | Activation PD-1 | functional |
| CD278 | 162Dy |  | functional |
| CD279 | 170Er | T-cell co-inhibitory receptor/exhaustion | functional |
| CD28 | 161Dy | T-cell costimulation | functional |
| CD314 | 146Nd |  | functional |
| CD319 | 152Sm |  | functional |
| CD357 | 165Ho |  | functional |
| CD366 | 153Eu | Effector T cells, T-cell exhaustion | functional |
| CD39 | 113Cd | B cells, T cells | functional |
| CD57 | 196Pt |  | functional |
| CD69 | 160Gd | T-cell early activation | functional |
| GPRC5D | 155Gd |  | functional |
| GranzymeB | 163Dy | Activated T cells, NK cells | functional |
| GranzymeK | 142Nd |  | functional |
| IgK | 195Pt | Myeloma cells | functional |
| IgL | 151Eu | B cells, Myeloma cells | functional |
| Tigit | 150Nd |  | functional |
| CD127 | 148Nd | Activated and regulatory T cells | phenotypic |
| CD138 | 145Nd | Plasma cells, multiple myeloma cells | phenotypic |
| CD14 | 171Yb | Monocytes, macrophages | phenotypic |
| CD15 | 164Dy | Granulocytes | phenotypic |
| CD16 | 209Bi | Proinflammatory monocytes, NK subset, granulocytes | phenotypic |
| CD161 | 147Sm |  | phenotypic |
| CD185 | 174Yb |  | phenotypic |
| CD19 | 143Nd | B lymphocytes | phenotypic |
| CD25 | 169Tm | Activated and regulatory T lymphocytes | phenotypic |
| CD27 | 167Er | Memory B lymphocytes, T lymphocytes | phenotypic |
| CD3 | 194Pt | T lymphocytes | phenotypic |
| CD34 | 115In |  | phenotypic |
| CD38 | 172Yb | Activation, plasma cells | phenotypic |
| CD4 | 111Cd | T-helper lymphocytes | phenotypic |
| CD45RA | 154Sm | Naive T lymphocytes | phenotypic |
| CD45RO | 149Sm | Memory T lymphocytes | phenotypic |
| CD56 | 176Yb | NK and NKT cells | phenotypic |
| CD8 | 116Cd | Cytotoxic T lymphocytes | phenotypic |
| CX3CR1 | 114Cd |  | phenotypic |
| CD45 | 89Y | Leukocytes | reference |
| CD66b | 139La | Granulocytes | reference |

**Data acquisition and normalization**

Data were acquired in FCS file format and normalized using CyTOF Software (v7.0.8493) for Stand-Alone Processing Workstations (Standard Bio Tools). Channel intensities were normalized with calibration beads and arcsinh-transformed with a cofactor of five. Manual gating was performed using Cytobank software. For downstream analysis, only samples with >10000 events in the “01_157Gd low-casp 3 low” (live gate) were included. A hypothesis-generating approach was then applied to detect data trends using in-house scripts in R 4.0.2.

**Quality control**

Quality control was performed using HilbertSimilarity distance (Abraham Y, et al. <https://zenodo.org/record/3557362#.YmayIdrMJPY>), Earth Mover’s Distance (7), and marker enrichment modelling (8). These metrics assessed potential batch effects at the sample, cluster, and marker levels as well as the overall quality of the samples and marker staining.

**Batch effect correction**

CyTOF data was generated and processed in different batches, introducing technical variation as measured using the above quality metrics. This variation was corrected using cyCombine (v0.2.15) (9) with the default parameters using the condition of the patient as covariate. Effectiveness of the correction was confirmed using HilbertSimilarity distance and Earth Mover’s Distance. Control-sample clustering was also used to confirm appropriate correction, where needed.

**Cell clustering and annotation**

Unsupervised clustering based on the cyCombine corrected values for the phenotypic, functional and reference markers was performed using a self-organizing map (SOM) in combination with consensus clustering (FlowSOM) (R package 2.5.6) (10). Cells were clustered in a 18x18 SOM grid. The output was visualized in a minimum-spanning tree (MST). The clusters on this MST were then grouped into metaclusters and marker expression in each metacluster was manually reviewed to identify known immune cell subsets, and subpopulations were further refined and annotated.

**Statistical analysis**

Prior to the statistical abundance analysis, plasma cells and granulocytes as well as cells without clear annotation were removed from the analysis to make the composition and starting material similar to the ADTseq data. Differences in population sizes were assessed by fitting a generalized linear mixed-effects model for the negative binomial family with the following formula: N ~ Condition + BatchID + offset(log(Total)), where N corresponds to the population size; Condition was HV, MGUS, SMM, or MM; and BatchID to account for technical variation introduced by the batch. These differences were assessed for all cell populations’ definitions as defined by the metacluster annotations. The contrasts that were included in the analysis were: (MGUS-HV), (SMM-HV), (MM-HV), (SMM-MGUS), (MM-MGUS), (MM-SMM).

Multiple testing correction was performed per population definition at two levels: over all contrasts, using Tukey, and over all populations using the FDR. Results were flagged as significant when the corrected p-value was smaller than 0.05, and the absolute estimated change was bigger than 1.

**References**

1. Aran D, Looney AP, Liu L, Wu E, Fong V, Hsu A, et al. Reference-based analysis of lung single-cell sequencing reveals a transitional profibrotic macrophage. Nat Immunol. 2019;20(2):163-72.

2. Robinson MD, McCarthy DJ, Smyth GK. edgeR: a Bioconductor package for differential expression analysis of digital gene expression data. Bioinformatics. 2009;26(1):139-40.

3. Zavidij O, Haradhvala NJ, Mouhieddine TH, Sklavenitis-Pistofidis R, Cai S, Reidy M, et al. Single-cell RNA sequencing reveals compromised immune microenvironment in precursor stages of multiple myeloma. Nat Cancer. 2020;1(5):493-506.

4. de Jong MME, Kellermayer Z, Papazian N, Tahri S, Hofste op Bruinink D, Hoogenboezem R, et al. The multiple myeloma microenvironment is defined by an inflammatory stromal cell landscape. Nat Immunol. 2021;22(6):769-80.

5. Pilcher WC, Yao L, Gonzalez-Kozlova E, Pita-Juarez Y, Karagkouni D, Acharya CR, et al. A single-cell atlas characterizes dysregulation of the bone marrow immune microenvironment associated with outcomes in multiple myeloma. bioRxiv. 2024.

6. Skerget S, Penaherrera D, Chari A, Jagannath S, Siegel DS, Vij R, et al. Comprehensive molecular profiling of multiple myeloma identifies refined copy number and expression subtypes. Nat Genet. 2024;56(9):1878-89.

7. Qiu P. Inferring phenotypic properties from single-cell characteristics. PLoS One. 2012;7(5):e37038.

8. Diggins KE, Greenplate AR, Leelatian N, Wogsland CE, Irish JM. Characterizing cell subsets using marker enrichment modeling. Nat Methods. 2017;14(3):275-8.

9. Pedersen CB, Dam SH, Barnkob MB, Leipold MD, Purroy N, Rassenti LZ, et al. cyCombine allows for robust integration of single-cell cytometry datasets within and across technologies. Nat Commun. 2022;13(1):1698.

10. Van Gassen S, Callebaut B, Van Helden MJ, Lambrecht BN, Demeester P, Dhaene T, et al. FlowSOM: Using self-organizing maps for visualization and interpretation of cytometry data. Cytometry A. 2015;87(7):636-45.
